# Fecal metagenomic profiles in subgroups of patients with myalgic encephalomyelitis/chronic fatigue syndrome

**DOI:** 10.1186/s40168-017-0261-y

**Published:** 2017-04-26

**Authors:** Dorottya Nagy-Szakal, Brent L. Williams, Nischay Mishra, Xiaoyu Che, Bohyun Lee, Lucinda Bateman, Nancy G. Klimas, Anthony L. Komaroff, Susan Levine, Jose G. Montoya, Daniel L. Peterson, Devi Ramanan, Komal Jain, Meredith L. Eddy, Mady Hornig, W. Ian Lipkin

**Affiliations:** 1 Center for Infection and Immunity, Columbia University Mailman School of Public Health, 722 W 168th Street 17th Floor, New York,, NY 10032 USA; 2 Fatigue Consultation Clinic, Salt Lake City, UT 84102 USA; 3 Institute for Neuro-Immune Medicine, College of Osteopathic Medicine, Nova Southeastern University, Fort Lauderdale, FL 33314 USA; 4 Brigham and Women’s Hospital, Harvard Medical School, Boston, MA 02115 USA; 5 Levine Clinic, New York, NY 10021 USA; 6 Stanford University, Palo Alto, CA 94305 USA; 7 Sierra Internal Medicine at Incline Village, Incline Village, NV 89451 USA; 8 Ayasdi, Inc., Menlo Park, CA 94025 USA; 9 Miami VA Medical Center, Miami, FL 33125 USA

**Keywords:** Myalgic encephalomyelitis, Chronic fatigue syndrome, Microbiota-gut-brain axis, Metagenomic, Topological data analysis, Irritable bowel syndrome, Metabolic pathway

## Abstract

**Background:**

Myalgic encephalomyelitis/chronic fatigue syndrome (ME/CFS) is characterized by unexplained persistent fatigue, commonly accompanied by cognitive dysfunction, sleeping disturbances, orthostatic intolerance, fever, lymphadenopathy, and irritable bowel syndrome (IBS). The extent to which the gastrointestinal microbiome and peripheral inflammation are associated with ME/CFS remains unclear. We pursued rigorous clinical characterization, fecal bacterial metagenomics, and plasma immune molecule analyses in 50 ME/CFS patients and 50 healthy controls frequency-matched for age, sex, race/ethnicity, geographic site, and season of sampling.

**Results:**

Topological analysis revealed associations between IBS co-morbidity, body mass index, fecal bacterial composition, and bacterial metabolic pathways but not plasma immune molecules. IBS co-morbidity was the strongest driving factor in the separation of topological networks based on bacterial profiles and metabolic pathways. Predictive selection models based on bacterial profiles supported findings from topological analyses indicating that ME/CFS subgroups, defined by IBS status, could be distinguished from control subjects with high predictive accuracy. Bacterial taxa predictive of ME/CFS patients with IBS were distinct from taxa associated with ME/CFS patients without IBS. Increased abundance of unclassified *Alistipes* and decreased *Faecalibacterium* emerged as the top biomarkers of ME/CFS with IBS; while increased unclassified *Bacteroides* abundance and decreased *Bacteroides vulgatus* were the top biomarkers of ME/CFS without IBS. Despite findings of differences in bacterial taxa and metabolic pathways defining ME/CFS subgroups, decreased metabolic pathways associated with unsaturated fatty acid biosynthesis and increased atrazine degradation pathways were independent of IBS co-morbidity. Increased vitamin B6 biosynthesis/salvage and pyrimidine ribonucleoside degradation were the top metabolic pathways in ME/CFS without IBS as well as in the total ME/CFS cohort. In ME/CFS subgroups, symptom severity measures including pain, fatigue, and reduced motivation were correlated with the abundance of distinct bacterial taxa and metabolic pathways.

**Conclusions:**

Independent of IBS, ME/CFS is associated with dysbiosis and distinct bacterial metabolic disturbances that may influence disease severity. However, our findings indicate that dysbiotic features that are uniquely ME/CFS-associated may be masked by disturbances arising from the high prevalence of IBS co-morbidity in ME/CFS. These insights may enable more accurate diagnosis and lead to insights that inform the development of specific therapeutic strategies in ME/CFS subgroups.

**Electronic supplementary material:**

The online version of this article (doi:10.1186/s40168-017-0261-y) contains supplementary material, which is available to authorized users.

## Background

Myalgic encephalomyelitis/chronic fatigue syndrome (ME/CFS) is an unexplained, chronic debilitating disorder that in the USA alone is estimated to affect up to 2.5 million people, with annual costs of $24 billion [1, 2]. Diagnosis is based on the presence of three of the following symptoms: (1) fatigue impairing an individual’s ability to engage in occupational, educational, social, or personal activities for at least 6 months; (2) post-exertional malaise and unrefreshing sleep; and (3) at least one of the following two symptoms: cognitive impairment and orthostatic intolerance [1]. Some patients report a prodrome with fever, sore throat, and lymphadenopathy [3]. Thirty-five to 90% of ME/CFS subjects report abdominal discomfort consistent with irritable bowel syndrome (IBS) [4–6].

Bacteria, their metabolites, and the host molecules they influence are participants in bidirectional communication pathways linking the gut and the central nervous system (CNS) [7, 8]. Intestinal dysbiosis can dysregulate local physiology (as in IBS) and immunological circuits [9] as well as cognition and mood [10, 11].

Culture-based and 16S ribosomal RNA (rRNA) gene sequencing studies of stool bacteria have revealed evidence of dysbiosis (an imbalance of intestinal bacterial populations) in ME/CFS [12–14]. Altered plasma metabolites have been identified that distinguish ME/CFS patients from healthy controls. At least some of these metabolites are products of the intestinal microbiome [15, 16]. Here, we complement and extend this work in a cohort of 50 ME/CFS and 50 healthy controls using shotgun metagenomic sequencing (SMS), metabolic pathway analysis, and linkage to clinical data and plasma immune profiles. We also employ a novel topological data analysis (TDA) platform that reveals relationships that may be overlooked with linear analytical models.

## Results

### Study population characteristics

Subjects included 50 ME/CFS cases and 50 healthy controls recruited at four sites across the USA (New York, NY; Salt Lake City, UT; Incline Village, NV; and Miami, FL) who met the 1994 CDC Fukuda [17] and the 2003 Canadian consensus criteria for ME/CFS [18]. Subject demographics are shown in Table 1. Cases included 41 female and 9 male ME/CFS patients (mean age 51.1 years; standard error of the mean (SEM) 1.6). Controls included 41 female and 9 male subjects (mean age 51.3 years; SEM 1.6). All case and control samples were collected between June 22, 2014, and October 27, 2014. IBS was diagnosed in 21 of 50 ME/CFS patients (42%) and none of 50 controls. Nine of 21 ME/CFS + IBS patients (43%) reported having IBS diagnosis prior to ME/CFS. No controls reported a diagnosis of IBS. Twenty-eight ME/CFS patients and 22 controls had a high body mass index (BMI) (>25 kg/m^2^).

**Table 1:** Characteristics of study cohort

		ME/CFS (*n* = 50)	Controls (*n* = 50)
Sex	Female	41	41
	Male	9	9
Age	Mean (±SEM)	51.081 (±1.607)	51.320 (±1.620)
	Median (range)	53.607 (20.493-66.500)	52.930 (21.040–67.869)
Race	White	49	48
	Asian	1	1
	Other	0	1
Ethnicity	Not Hispanic or Latino	46	45
	Hispanic or Latino	4	5
Site of Collection	New York, NY	14	14
	Salt Lake City, UT	14	15
	Sierra, NV	12	12
	Miami, FL	10	9
Season of Collection	Summer	27	26
	Fall	23	24
IBS Co-morbidity	With IBS	21	0
	Without IBS	29	50
BMI	High BMI (>25 kg/m^2^)	28	22
	Normal BMI (<25 kg/m^2^)	22	28
Duration of ME/CFS	Long duration (>3 years)	46	N/A
	Short duration (<3 years)	4	N/A

*ME/CFS* myalgic encephalomyelitis/chronic fatigue syndrome, *IBS* irritable bowel syndrome, *BMI* body mass index, *SEM* standard error of the mean, *N/A* not applicable

### TDA analysis of fecal microbiota, predicted bacterial metabolic pathways, plasma immune molecule profiles, and clinical features

Shotgun metagenomic sequencing of fecal samples was pursued to determine microbial composition (relative abundance of taxa) and infer bacterial metabolic pathways in the ME/CFS and control subjects. An average of 7 Gb of sequence per sample (from 100 bp, paired-end Illumina reads) was generated using high-throughput sequencing. Levels of plasma immune molecules were quantitated by immunoassay. We built a TDA network comprised of 100 samples (50 cases and 50 controls) and 1358 total variables. The variables consisted of the following elements: 574 representing the relative abundance of bacterial taxa; 586 representing metabolic pathways (131 superpathways and 455 individual metabolic pathways); 61 reflecting levels of each plasma immune molecule in the assay; 80 representing symptoms (health questionnaire items); and 57 co-morbidities and demographic variables.

Relationships among these datasets were analyzed using TDA (AYASDI software) to identify multidimensional networks and the individual factors (microbial, metabolic pathways, immune molecules, and clinical variables) that distinguish those networks.

**Fig. 1:**
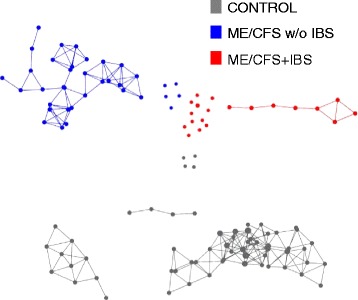
Topological data analysis (TDA) reveals altered metagenomic profiles in ME/CFS. Metagenomic data including bacterial composition predicted bacterial metabolic pathways, plasma immune profiles and symptom severity scores were analyzed by using TDA (AYASDI software) to define multidimensional subgroups. TDA of variance-normalized Euclidean distance metric with four lenses [neighborhood lenses (NL1 and NL2), ME/CFS, and IBS diagnosis] revealed that ME/CFS samples formed distinct networks separately from controls. The controls grouped more tightly than the ME/CFS patients. IBS co-morbidity was identified as the strongest driving factor in the separation of metagenomic and immune profile of ME/CFS individuals. *Dots* that are not connected to the networks represent outliers. Metagenomic data including bacterial composition and inferred metabolic pathways, plasma immune profiles, and health symptom severity scores were integrated for topological data analysis (TDA) using the AYASDI platform (Ayasdi, Menlo Park, California). AYASDI represents high-dimensional, complex biological data sets as a structured 3-dimensional network [56]. Each node in the network comprises one or more subject(s) who share variables in multiple dimensions. *Lines* connect network nodes that contain shared data points. Unlike traditional network models where a single sample makes a single node, the size of a node in the topological network was proportional to the number of variables with a similar profile. We built a network comprised of 100 samples and 1358 variables (574 variables representing bacterial relative abundance at different taxonomic levels, 61 variables reflecting levels of each immune molecule in the assay, 586 variables representing metabolic pathways, 80 variables representing different ME/CFS fatigue, and other symptom score/health questionnaire items and information on co-morbidities, and demographic variables). All variables were weighted equally. Variance-normalized Euclidean distance method was used as the distance metric; a range of filter lenses (neighborhood lens 1 and 2, ME/CFS, and IBS diagnosis) was used to identify networks

The ME/CFS subjects formed separate topological networks from the control subjects in TDA (Fig. 1). IBS co-morbidity was the strongest driving factor in the separation of metagenomics in ME/CFS. TDA revealed differences in bacterial taxa and metabolic pathways between ME/CFS, ME/CFS + IBS, and ME/CFS without IBS vs. controls (Additional file 1: Table S1A). At the family level, the relative abundances of *Lachnospiraceae* and *Porphyromonadaceae* were lower in the ME/CFS (both with and without IBS) compared to the controls, whereas the relative abundance of the family *Clostridiaceae* was higher. At the genus level, the abundances of *Dorea*, *Faecalibacterium*, *Coprococcus*, *Roseburia*, and *Odoribacter* were lower in the ME/CFS compared to the controls, whereas abundances of *Clostridium* and *Coprobacillus* were higher. The 12 bacterial species driving the differences between the ME/CFS and control groups were *Faecalibacterium prausnitzii*, *Faecalibacterium* cf., *Roseburia inulinivorans*, *Dorea longicatena*, *Dorea formicigenerans*, *Coprococcus catus*, *Odoribacter splanchnicus*, *Ruminococcus obeum*, and *Parabacteroides merdae* (all decreased in ME/CFS) and *Clostridium asparagiforme*, *Clostridium symbiosum*, and *Coprobacillus bacterium* (all increased in ME/CFS).

Intragroup variability in the control group was smaller than in the ME/CFS group (Fig. 1). The TDA-based microbial taxa and metabolic pathways distinguishing between ME/CFS + IBS and ME/CFS without IBS vs. control are shown in Additional file 1: Table S1A. ME/CFS + IBS had decreased representation from the *Proteobacteria* phylum, *C. catus*, and *F. prausnitzii* species and increased representation from the *Clostridiaceae* family compared to the controls. Superpathway analysis showed alteration in heme biosynthesis, carboxylates, amino acid (AA), and polyamine metabolism. In the ME/CFS without the IBS group, the difference was driven by the increased abundance of members of the *Clostridiaceae* family, the *Clostridium*, and *Pseudoflavonifractor* genera, and the decreased abundance of members of the *Porphyromonadaceae* family and *Odoribacter and Parabacteroides* genera. The bacterial species driving the differences between the ME/CFS without IBS and control groups were *D. formicigenerans*, *C. catus*, *Blautia hansenii*, *and Parabacteroides distasonis* (all decreased in the ME/CFS without IBS) and unclassified *Bacteroides*, *D. longicatena*, *Ruminococcus gnavus*, *C. symbiosum*, *Eggerthella lenta*, *Pseudoflavonifractor capillosus*, *C. bacterium*, *Clostridium* cf. and *scindens* (all increased in the ME/CFS without IBS). The ME/CFS subgroup comparison between the ME/CFS + IBS and ME/CFS without IBS is shown in Additional file 1: Table S1A. Higher KS (Kolmogorov-Smirnov) scores were found for bacterial taxa and metabolic pathways distinguishing the ME/CFS + IBS group from controls. Comparisons involving the ME/CFS + IBS subgroup (vs. controls and vs. ME/CFS without IBS) showed stronger associations with bacterial taxa and metabolic pathways than any of the other comparisons.

Four networks defined by IBS co-morbidity and BMI (Additional file 2: Figure S1, Additional file 1: Table S1B) were associated with a distinct metagenomic and immune profile.

### ME/CFS and ME/CFS subgroups are associated with an altered microbial composition

**Fig. 2:**
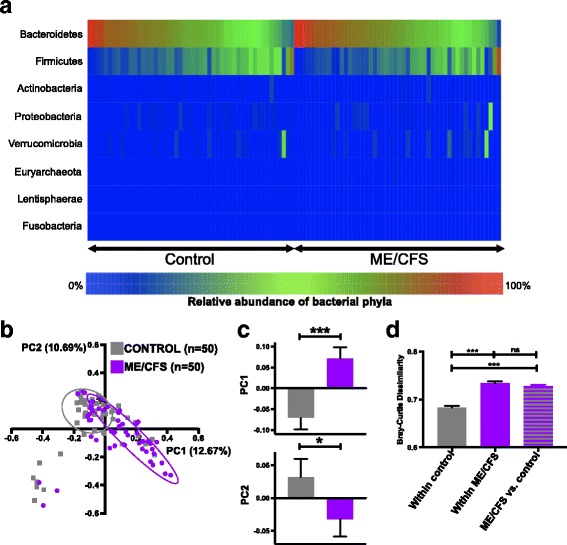
The altered microbial profile of ME/CFS compared to controls. a Heatmap representing the relative abundance of phyla in ME/CFS and control subjects. *Bacteroidetes* and *Firmicutes* were the two dominant phyla in both ME/CFS and control individuals. The heatmap represents the individual values (relative abundances) of bacterial phyla as colors where *blue* is the minimum percentage (0%) and *red* is the maximum percentage (100%). **b** Principal coordinate analysis (PCoA) based on the Bray-Curtis dissimilarity among species-level relative abundance distributions showed overlap between ME/CFS and control subject microbiota. **c** Bar charts showing significant separation of ME/CFS and controls along the first two PCs (PC1 explaining 12.67% of the variance, ***p < 0.001; PC2 explaining 10.69% of the variance, *p = 0.030). PCoA coordinates were compared as continuous variables with nonparametric Mann-Whitney *U* test. **d** Bar chart showing the BC dissimilarity within control subjects, within ME/CFS subjects, and between ME/CFS vs. control subjects. BC dissimilarity values were compared as continuous variables with nonparametric Mann-Whitney *U* test; *error bars* show the mean with SEM (standard error of the mean). *p < 0.05; ***p < 0.001. *ns* not significant

Compositional taxonomic analysis based on metagenomic sequencing indicated that the two dominant phyla in both ME/CFS and control individuals were *Bacteroidetes* (64.9 and 63.4%, respectively) and *Firmicutes* (26.6 and 29.7%, respectively) (Fig. 2a). Combined, *Bacteroidetes* and *Firmicutes* accounted for a mean relative abundance of 91.5% in ME/CFS cases and 93.1% in controls. The other phyla (*Actinobacteria*, *Proteobacteria*, *Verrucomicrobia*, *Euryarchaeota*, *Lentisphaerae*, and *Fusobacteria*) were represented at low relative abundance (mean relative abundance <5%) in samples.

Principal coordinate analysis (PCoA) based on the species-level Bray-Curtis [19] dissimilarity revealed overlap between the ME/CFS and control subjects (Fig. 2b). However, the ME/CFS subjects overall varied from the controls in the first two principal coordinates [20], accounting for 25% of the total variance (Fig. 2c: PC1 12.67%, *p* < 0.001; PC2 10.69%, *p* = 0.03). Within control subjects, BC dissimilarity was significantly lower than within ME/CFS subjects, consistent with our findings based on TDA analysis (Fig. 1) and suggests greater variability in the ME/CFS microbiota (Fig. 2d). The between-group (ME/CFS vs. control) BC dissimilarity comparisons were higher than the within-control comparisons (*p* < 0.001) but was not higher than the within ME/CFS comparisons (Fig. 2d). Together, these data provide evidence of greater variability in the microbiota of ME/CFS patients.

**Fig. 3:**
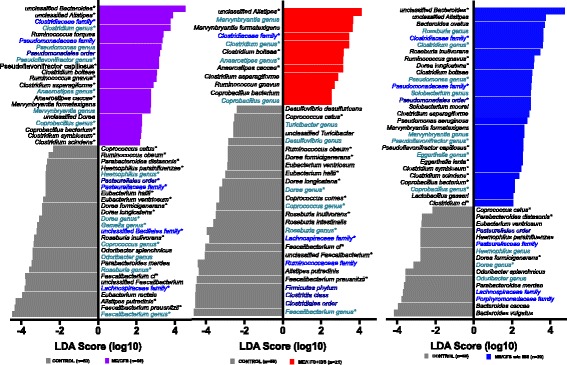
Bacterial taxa differentiate ME/CFS and ME/CFS subgroups (ME/CFS + IBS and ME/CFS without IBS) compared to controls. Histogram showing the log-transformed LDA scores computed with LEfSe for bacterial taxa differentially abundant between ME/CFS groups and controls. Positive LDA score indicates enrichment in **a** ME/CFS, **b** ME/CFS + IBS, and **c** ME/CFS without IBS vs. controls. Negative LDA indicates enrichment in control subjects (reduced in ME/CFS subgroups). The LDA score indicates the effect size and ranking of each bacterial taxon. Names of different taxonomic categories (genus, family, or order) are indicated with shades of *blue*; species names are written in *black*. An alpha value of 0.05 for the Kruskal-Wallis test and a log-transformed LDA score of 2.0 were used as thresholds for significance in LEfSe analyses. *Asterisks* next to taxa names indicate significant differences that were also found for a given taxa based on nonparametric Mann-Whitney *U* test with Benjamini correction (adjusted *p* < 0.2)

Metagenomic biomarker discovery (linear discriminant analysis effect size (LEfSe)) identified 22 bacterial taxa enriched in ME/CFS and 27 enriched in controls (Fig. 3a). Based on nonparametric Mann-Whitney *U* test with Benjamini-Hochberg correction (*p* < 0.05 and adjusted *p* < 0.2), 41 bacterial species, genera, families, or orders differed between the ME/CFS and control groups (Additional file 1: Table S2A). Thirty-seven bacterial taxa differentiated ME/CFS from controls by both statistical methods. At the bacterial order and of family levels, the relative abundances of members of the order *Pasteurellales* and of the families of *Lachnospiraceae*, unclassified *Bacillales* and *Pasteurellaceae* were lower in the ME/CFS patients than in the controls, whereas the relative abundance of members of the family *Clostridiaceae* was higher in the ME/CFS. At the genus levels, the abundances of members of the genera *Faecalibacterium*, *Roseburia*, *Coprococcus*, *Gemella*, *Dorea*, and *Haemophilus* were lower in the ME/CFS, whereas the abundances of the genera *Clostridium*, Pseudoflavonifractor, *Anaerostipes* and *Coprobacillus* were higher in the ME/CFS. The bacterial species driving the differences between the ME/CFS and control groups were *F. prausnitzii*, *Alistipes putredinis*, *Faecalibacterium* cf., *R. inulinivorans*, *D. longicatena*, *D. formicigenerans*, *Eubacterium ventriosum*, *Eubacterium hallii*, *Haemophilus parainfluenzae*, *P. distasonis*, *R. obeum* and *C. catus* (all decreased in ME/CFS) and unclassified *Bacteroides*, *unclassified Alistipes*, *P. capillosus*, *Clostridium bolteae*, *R. gnavus*, *C. asparagiforme*, *Anaerostipes caccae*, *C. bacterium*, *C. symbiosum*, and *C. scindens* (all increased in ME/CFS). Species in *Faecalibacterium*, *Roseburia*, *Dorea*, *Coprococcus*, *Clostridium*, *Ruminococcus*, and *Coprobacillus* species were significantly different in the ME/CFS patients compared to the controls by TDA, LEfSe, and nonparametric testing (Fig. 3a, Additional file 1: Table S1A and Additional file 1: Table S2A).

Evidence from TDA that IBS was linked to differences in disease severity, microbiota, and immune profiles led us to stratify the patient cohort into ME/CFS + IBS and ME/CFS without IBS and test for group-specific differences in the microbiota using linear statistical models.

In comparisons of the ME/CFS + IBS and control groups, LEfSe identified 12 bacterial species, genera, or families enriched in the ME/CFS + IBS and 26 bacterial taxa enriched in the controls (Fig. 3b). Based on nonparametric Mann-Whitney *U* test with Benjamini-Hochberg correction (adjusted *p* < 0.2), 21 bacterial taxa differed between the ME/CFS + IBS and control groups (Additional file 1: Table S2B). The two statistical methods yielded results with overlap in all 21 bacterial taxa. Differences were driven by increases in representatives of the *Clostridiaceae* family, *Clostridium* and *Anaerostipes* genera; and decreases in representatives of the *Lachnospiraceae* family, *Faecalibacterium*, *Roseburia*, *Coprococcus*, and *Dorea* genera. The 13 bacterial species driving the differences between ME/CFS + IBS and controls were *F. prausnitzii*, *F. cf.*, unclassified *Faecalibacterium*, *R. inulinivorans*, *C. comes*, *D. longicatena*, *E. hallii*, *D. formicigenerans*, *R. obeum*, and *C. catus* (each of which was decreased in the ME/CFS + IBS) and unclassified *Alistipes*, *C. bolteae*, and *A. caccae* (which were increased in the ME/CFS + IBS).

In the ME/CFS without IBS, LEfSe identified 29 bacterial species, genera or families enriched in ME/CFS without IBS and 16 bacterial taxa enriched in controls (Fig. 3c). Based on nonparametric Mann-Whitney *U* test with Benjamini-Hochberg correction (adjusted *p* < 0.2), 22 bacterial taxa differed between the ME/CFS without IBS and control groups (Additional file 1: Table S2B). Twenty-one bacterial taxa showed overlap between the two statistical methods. The differences in ME/CFS without IBS were driven by the increased abundance of members of *Pseudomonadales* order, the *Clostridiaceae* and *Pseudomonadaceae* family, and the *Clostridium*, *Pseudomonas*, *Pseudoflavonifractor*, *Eggerthella*, and *Coprobacillus* genera and the decreased abundance of members of the *Dorea* genus. The 13 bacterial species driving the differences between the ME/CFS without IBS and control groups were *D. formicigenerans*, *C. catus*, and *P. distasonis*, (all decreased in ME/CFS without IBS) and unclassified *Bacteroides*, *R. gnavus*, *D. longicatena*, *P. capillosus*, *E. lenta*, *C. symbiosum* and *scindens*, *C. bacterium*, and *Clostridium* cf. (all increased in ME/CFS without IBS).

**Fig. 4:**
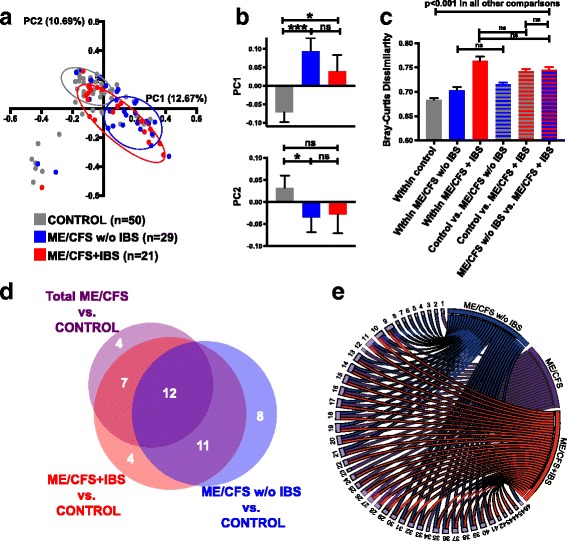
Distinct microbial profiles of ME/CFS + IBS and ME/CFS without IBS compared to controls. **a** Principal coordinate analysis (PCoA) analysis based on the BC dissimilarity with ME/CFS stratified by IBS co-morbidity. **b** Bar charts showing separation of ME/CFS with and without IBS compared to controls along PC1 (top panel); only ME/CFS without IBS showed significant separation along PC2 (*bottom panel*). **c** Bar chart showing the mean BC dissimilarity within control subjects, within ME/CFS without IBS and within ME/CFS with IBS subjects, as well as, between control vs. ME/CFS without IBS, between control vs. ME/CFS with IBS, and between ME/CFS without IBS vs. ME/CFS with IBS subjects. PCoA coordinates and BC dissimilarity values were compared as continuous variables with nonparametric Mann-Whitney *U* test; error bars show the SEM (standard error of the mean). *p < 0.05; ***p < 0.001. *ns* not significant. **d**, **e** Unique and overlapping bacterial species differentiate ME/CFS and ME/CFS IBS subgroups from control subjects. **d** Proportional Venn diagram showing the number of unique and overlapping bacterial species that differentiate ME/CFS groups from control subjects. **e** Circular visualization (Circos) showing the individual bacterial species and their unique or overlapping relationship between the total ME/CFS group, the ME/CFS without IBS group, and the ME/CFS with IBS group. Unique to total ME/CFS: *Gemella* unclassified (*1*), *Streptococcus peroris* (*12*), *Eubacterium rectale* (*23*), and *Ruminococcus torques* (*34*). Unique to ME/CFS without IBS: *Bacteroides caccae* (*2*), *Bacteroides ovatus* [64], *Bacteroides vulgatus* (*4*), *Lactobacillus gasseri* (*5*), *Streptococcus infantis* (*6*), *Solobacterium moorei* (*7*), *Pseudomonas aeruginosa* (*8*), and *Eggerthella lenta* (*46*). Unique to ME/CFS + IBS: *Butyrivibrio* unclassified (*42*), *Coprococcus comes* (*43*), *Roseburia intestinalis* (*44*), and *Desulfovibrio desulfuricans* (*45*). Overlapping between all three comparisons (total ME/CFS, ME/CFS without IBS and ME/CFS + IBS groups vs. control): *Alistipes* unclassified (*9*), *Clostridium asparagiforme* (*10*), *Clostridium bolteae* (*11*), *Eubacterium ventriosum* (*13*), *Coprococcus catus* (*14*), *Dorea formicigenerans* (*15*), *Dorea longicatena* (*16*), *Marvinbryantia formatexigens* (*17*), *Roseburia inulinivorans* (*18*), *Ruminococcus gnavus* (*19*), *Subdoligranulum variabile* (*20*),and *Coprobacillus bacterium* (*21*). Overlapping between total ME/CFS and ME/CFS + IBS groups compared to controls: *Alistipes putredinis* (*22*), *Eubacterium hallii* (*24*), *Anaerostipes caccae* (*25*), *Faecalibacterium* cf. (*26*), *Faecalibacterium prausnitzii* (*27*), *Faecalibacterium* unclassified (*28*), and *Ruminococcus obeum* (*29*). Overlapping between ME/CFS without IBS and ME/CFS + IBS groups compared to controls: *Bacteroides* unclassified (*30*), *Odoribacter splanchnicus* (*31*), *Parabacteroides distasonis* (*32*), *Parabacteroides merdae* (*33*), *Clostridium* cf. (*35*), *Clostridium scindens* (*36*), *Clostridium symbiosum* (*37*), *Pseudoflavonifractor capillosus* (*38*), *Blautia hansenii* (*39*), *Dorea* unclassified (*40*), and *Haemophilus parainfluenzae* (*41*)

PCoA analysis based on the BC dissimilarity with ME/CFS stratified by IBS co-morbidity again showed overlap in controls and ME/CFS (Fig. 4a). Both the ME/CFS + IBS and ME/CFS without IBS individuals separated along PC1 compared to the controls (Fig. 4b: PC1 12.67%, ME/CFS + IBS vs. control *p* < 0.05, ME/CFS without IBS vs. control *p* < 0.001); however, only ME/CFS without IBS showed separation along PC2 (PC2 10.69%, ME/CFS without IBS vs. control *p* = 0.030). While controls had significantly lower within-group dissimilarity compared to within-group dissimilarities of both the ME/CFS without IBS and ME/CFS + IBS, ME/CFS + IBS had the highest within-group dissimilarity (Fig. 4c). Between-group comparisons demonstrated that there was lower dissimilarity between the controls and the ME/CFS without IBS group than there was between the controls and the ME/CFS group with IBS. Comparison between ME/CFS without IBS vs. ME/CFS with IBS showed higher dissimilarity than control vs. ME/CFS without IBS but similar dissimilarity as between control vs. ME/CFS with IBS. Thus, there was as much dissimilarity between the two subgroups of ME/CFS defined by IBS co-morbidity as between control and ME/CFS with IBS and even greater dissimilarity between the subgroups of IBS. Together, these results suggest that the ME/CFS subjects have greater variation between their microbiota than the control subjects and that ME/CFS subjects with IBS have the greatest within group variation.

Proportional Venn and circular visualization diagrams were used to display the overlapping and differentiating bacterial taxa between the controls and each of the ME/CFS groups (total ME/CFS, ME/CFS + IBS, and ME/CFS without IBS) (Fig. 4d, e). The proportional Venn diagram (Fig. 4d) showed that the relative abundance of 11 bacterial species distinguished both ME/CFS + IBS and ME/CFS without IBS from the controls. The relative abundance of 19 bacterial species distinguished the ME/CFS subjects without IBS from the controls, but did not help to differentiate the total ME/CFS group from the control group. Circular visualization (Fig. 4e) showed both the distinct and overlapping bacterial taxa whose abundance differentiated the total ME/CFS group, the ME/CFS + IBS group, and the ME/CFS group without IBS from the control group.

We compared the number of bacterial species among the ME/CFS diagnostic groups and the controls. A total of 363 bacterial species were identified in the entire cohort. The number of species identified was similar in ME/CFS patients and controls (average number of bacterial species ± the standard error of the mean: ME/CFS 74.24 ± 1.67; control 77.5 ± 2.07). We also did not find differences in the number of bacterial species when comparing subgroups based on IBS status (average number of bacterial species ± the standard error of the mean: ME/CFS + IBS 71.62 ± 2.26; ME/CFS without IBS 76.14 ± 2.34; control 77.5 ± 2.07).

### Bacterial species distinguish ME/CFS and ME/CFS with IBS from healthy control subjects

We used three dimensionality reduction methods to further analyze the microbial communities that distinguish ME/CFS subgroups and controls: least absolute shrinkage and selection operation (LASSO), random forest (RF) and partial least squares (PLS) (Table 2). Bacterial species were selected for the predictive logistic regression model if they met criteria in the dimensionality reduction models. Species from the *Firmicutes* phylum were the chief determinants of ME/CFS group status. The relative abundance of four bacterial species (*C. catus*, *P. capillosus*, *D. formicigenerans*, and *F. prausnitzii*) distinguished the ME/CFS patients from the controls (ROC AUC = 0.831 and cross-validated AUC = 0.684). The addition of four more bacterial species (*C. asparigiforme*, *Sutterella wadsworthensis*, *A. putredinis*, and *Anaerotruncus colihominis*) improved predictive performance (ROC AUC = 0.893, cross-validated AUC = 0.745). This predictive selection model corresponds to our TDA, LEfSe, and nonparametric statistical findings of differences in *Coprococcus*, *Dorea*, *Faecalibacterium*, and *Clostridium* spp. in total ME/CFS and control group comparisons.

**Table 2:** Predictive selection model defined different bacterial composition in ME/CFS diagnostic groups

Diagnostic groups Selected bacterial species		In-sample	Cross-Validation			
		AUC	AUC	ER (%)	FP (%)	FN (%)
ME/CFS (50) vs. control (50)						
*[Firmicutes] Coprococcus catus*	↓	0.831	0.684	36.72	40.00	33.43
*[Firmicutes] Pseudoflavonifractor capillosus*	↑					
*[Firmicutes] Dorea formicigenerans*	↓					
*[Firmicutes] Faecalibacterium prausnitzii*	↓					
*[Firmicutes] Coprococcus catus*	↓	0.893	0.745	29.68	29.71	29.64
*[Firmicutes] Pseudoflavonifractor capillosus*	↑					
*[Firmicutes] Dorea formicigenerans*	↓					
*[Firmicutes] Faecalibacterium prausnitzii*	↓					
*[Firmicutes] Clostridium asparagiforme*	↑					
*[Proteobacteria] Sutterella wadsworthensis*	↓					
*[Bacteroidetes] Alistipes putredinis*	↓					
*[Firmicutes] Anaerotruncus colihominis*	↑					
ME/CFS + IBS (21) vs. control (50)						
*[Firmicutes] Faecalibacterium cf*	↓	0.771	0.571	33.09	18.57	69.38
*[Bacteroidetes] Bacteroides vulgatus*	↑					
*[Firmicutes] Faecalibacterium cf*	↓	0.923	0.687	25.59	18.43	43.48
*[Bacteroidetes] Bacteroides vulgatus*	↑					
*[Firmicutes] Faecalibacterium prausnitzii*	↓					
*[Bacteroidetes] Alistipes putredinis*	↓					
*[Firmicutes] Coprococcus catus*	↓					
*[Firmicutes] Faecalibacterium cf*	↓	1	0.791	24.31	18.28	39.40
*[Bacteroidetes] Bacteroides vulgatus*	↑					
*[Firmicutes] Faecalibacterium prausnitzii*	↓					
*[Bacteroidetes] Alistipes putredinis*	↓					
*[Firmicutes] Coprococcus catus*	↓					
*[Firmicutes] Anaerostipes caccae*	↑					
*[Firmicutes] Dorea formicigenerans*	↓					
*[Firmicutes] Anaerotruncus colihominis*	↑					
*[Firmicutes] Clostridium asparagiforme*	↑					
ME/CFS w/o IBS (29) vs. control (50)						
*[Bacteroidetes] Bacteroides caccae*	↓	0.775	0.589	40.67	25.29	66.30
*[Firmicutes] Pseudoflavonifractor capillosus*	↑					
*[Bacteroidetes] Bacteroides caccae*	↓	0.948	0.754	28.41	21.07	40.65
*[Firmicutes] Pseudoflavonifractor capillosus*	↑					
*[Bacteroidetes] Parabacteroides distasonis*	↓					
*[Bacteroidetes] Bacteroides fragilis*	↑					
*[Bacteroidetes] Prevotella buccalis*	↑					
*[Bacteroidetes] Bacteroides xylanisolvens*	↑					
*[Firmicutes] Dorea formicigenerans*	↓					
ME/CFS w/o IBS (29) vs. ME/CFS + IBS (21)						
*[Bacteroidetes] Bacteroides vulgatus*	↓	0.913	0.604	37.61	29.30	50.08
*[Bacteroidetes] Prevotella buccalis*	↑					
*[Firmicutes] Ruminococcus lactaris*	↑					
*[Firmicutes] Eubacterium hallii*	↑					
*[Firmicutes] Anaerotruncus colihominis*	↓					
*[Firmicutes] Faecalibacterium cf*	↑					
*[Firmicutes] Clostridium methylpentosum*	↑					
*[Bacteroidetes] Bacteroides vulgatus*	↓	0.956	0.604	41.51	27.32	62.80
*[Bacteroidetes] Prevotella buccalis*	↑					
*[Firmicutes] Ruminococcus lactaris*	↑					
*[Firmicutes] Eubacterium hallii*	↑					
*[Firmicutes] Anaerotruncus colihominis*	↓					
*[Firmicutes] Faecalibacterium cf*	↑					
*[Firmicutes] Clostridium methylpentosum*	↑					
*[Firmicutes] Faecalibacterium prausnitzii*	↑					
*[Proteobacteria] Klebsiella pneumoniae*	↓					
*[Bacteroidetes] Parabacteroides distasonis*	↓					
*[Bacteroidetes] Prevotella copri*	↑					

The arrows represent the increased or decreased relative abundance of bacterial species in total ME/CFS, ME/CFS + IBS, ME/CFS w/o IBS (compared to controls). In the last comparison, the arrow represents changes in the ME/CFS without IBS group (compared to ME/CFS with IBS). *ME/CFS* myalgic encephalomyelitis/chronic fatigue syndrome, *IBS* irritable bowel syndrome, *AUC* area under the curve, *ER* error rate, *FP* false positive, *FN* false negative

The relative abundance of distinct bacterial taxa defined ME/CFS patients with IBS. The relative abundance of just two bacterial species (*Faecalibacterium* cf. and *Bacteroides vulgatus*) distinguished the ME/CFS + IBS from the control subjects with a moderate degree of accuracy (ROC AUC = 0.771; cross-validated AUC = 0.571). Accuracy of the outcome prediction improved when nine additional bacterial species: *F.* cf., *F. prausnitzii*, *B. vulgatus*, *A. putredinis*, *C. catus*, *A. caccae*, *D. formicigenerans*, *A. colihominis*, and *C. asparagiforme* were added to the model (ROC AUC = 1, cross-validated AUC = 0.791).

Membership in the ME/CFS without IBS subgroup as compared with the control group was predicted by the relative abundance of *Bacteroides caccae*, *P. capillosus*, *P. distasonis*, *Bacteroides fragilis*, *Prevotella buccalis*, *Bacteroides xylanisolvens*, and *D. formicigenerans* (ROC AUC = 0.948, cross-validated AUC = 0.754). TDA-selected bacterial species of *Bacteroides*, *Clostridium*, *Pseudoflavonifractor*, and *Parabacteroides* were found in all statistical tests (LEfSe, nonparametric and reduction model tests) in the ME/CFS without IBS group compared to the controls. The decreased relative abundance of *Bacteroides vulgatus* distinguished the ME/CFS without IBS group from the ME/CFS + IBS group based on all statistical tests (TDA, LEfSe, nonparametric and reduction model tests).

ME/CFS patients with IBS were distinguished from ME/CFS patients without IBS by the relative abundance of eleven bacterial species (ROC AUC = 0.956, cross-validated AUC = 0.604).

### Distinct bacterial metabolic pathways in ME/CFS

**Fig. 5:**
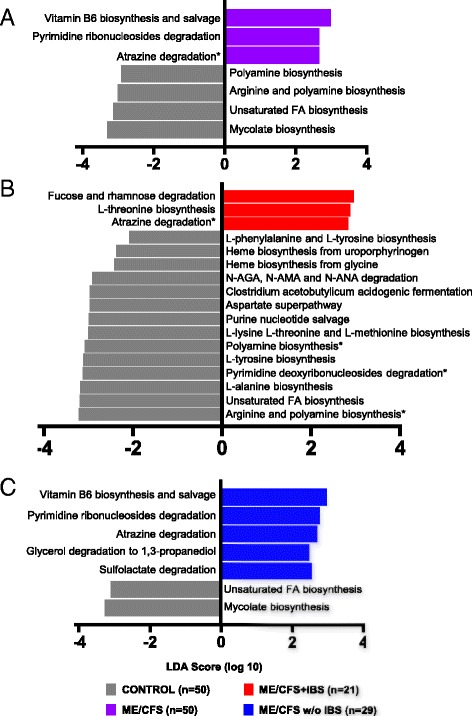
Altered bacterial metabolic pathways define ME/CFS and ME/CFS subgroup with IBS co-morbidity. Histogram of the log-transformed LDA scores computed with LEfSe for bacterial metabolic pathways found to be differentially abundant between ME/CFS groups and controls. Positive LDA score indicates enrichment of pathways in **a** ME/CFS, **b** ME/CFS + IBS, and **c** ME/CFS without IBS vs. controls. Negative LDA indicates enrichment of pathways in control subjects (reduced in ME/CFS groups). The LDA score indicates the effect size and ranking of each superpathway. An alpha value of 0.05 for the Kruskal-Wallis test and a log-transformed LDA score of 2.0 were used as thresholds for significance in LEfSe analyses. *Asterisks* next to pathways indicate significant differences were also found for a given pathway based on nonparametric Mann-Whitney *U* test with Benjamini-Hochberg correction (adjusted *p* < 0.2)

Bacterial metagenomic data were used to predict differences in functional metabolic pathways in the ME/CFS subgroups. Altogether, 455 individual bacterial metabolic pathways were identified and analyzed. In superpathway analyses (total 131 superpathways), LEfSe revealed that bacterial vitamin B6 biosynthesis and salvage, pyrimidine ribonucleoside degradation, and atrazine degradation were significantly enriched while bacterial pathways for the biosynthesis of arginine, polyamine, unsaturated fatty acid (FA), and mycolate were significantly reduced in the ME/CFS compared to the controls (Fig. 5a). The ME/CFS + IBS group had predicted enrichment in bacterial pathways for fucose, rhamnose, atrazine degradation and L-threonine biosynthesis, reduced heme, AA and polyamine biosynthesis, and reduced purine, pyrimidine, and unsaturated FA metabolism compared to the controls (Fig. 5b). In the ME/CFS without the IBS group, predicted bacterial pathways of vitamin B6 biosynthesis and salvage, pyrimidine ribonucleosides, atrazine, glycerol and sulfolactate degradation were increased, whereas unsaturated FA and mycolate biosynthesis were decreased compared to the controls (Fig. 5c). Nonparametric Mann-Whitney *U* test with Benjamini-Hochberg correction (adjusted *p* < 0.2) further supported findings from LEfSe showing enrichment in the pathway of atrazine degradation in both the ME/CFS and ME/CFS + IBS groups compared to the controls; predicted bacterial pathways of arginine, polyamine biosynthesis, and pyrimidine ribonucleoside degradation were reduced in the ME/CFS + IBS (Additional file 1: Table S3). Based on nonparametric Mann-Whitney *U* test with Benjamini-Hochberg correction (adjusted *p* < 0.2), ME/CFS showed altered representation of individual bacterial metabolic pathways linked to the tricarboxylic acid [21] cycle, alcohol and aromatic compound degradation, and FA/lipid metabolism (Additional file 1: Table S3). The ME/CFS + IBS group was associated with altered bacterial pathways for FA/lipid metabolism, aromatic compounds biosynthesis, and carbohydrate (CHO)/carboxylate degradation (Additional file 1: Table S3).

### Plasma cytokine concentrations do not distinguish ME/CFS groups

Immune profiling (Additional file 1: Table S4A) was performed to test for alterations in the ME/CFS and IBS subgroups. No significant findings were obtained after adjusting for multiple comparisons of all 61 cytokines. However, prior to adjustment, TNF-α was increased in the ME/CFS cases compared to the controls (Additional file 1: Table S4B) and plasma levels of leptin, CSF-2, CXCL-8, and TNF-α were higher in the ME/CFS + IBS patients than the controls (Additional file 1: Table S4B). The ME/CFS patients without IBS had a pre-adjustment trend toward increased TNF-α compared to controls.

Unsupervised hierarchical clustering was used to visualize the variation in plasma cytokine levels between the total ME/CFS, ME/CFS + IBS, ME/CFS without IBS, and controls. Although the clusters did distinguish a range of cytokine profiles in individuals (ranging from high to low cytokine profiles), there was no distinct clustering observed between disease groups (Additional file 2: Figure S2).

A predictive logistic regression model restricted solely to immune data showed little accuracy in distinguishing between the ME/CFS diagnostic groups and controls (total ME/CFS, ME/CFS + IBS, or ME/CFS without IBS vs. controls; data not shown).

### Correlations of symptom severity scores with bacterial species abundance and predicted bacterial metabolic pathways in ME/CFS and ME/CFS subgroups

We investigated whether the relative abundance of individual bacterial species, defined by their association with ME/CFS and ME/CFS subgroups (Fig. 3, Additional file 1: Table S2), correlated with Short Form 36 Health Survey (SF-36) and the Multidimensional Fatigue Inventory (MFI). Additional file 1: Table S5 shows the significant correlations of bacterial species with disease severity scores in all ME/CFS, ME/CFS + IBS, or ME/CFS without IBS cases.

Among all ME/CFS cases, the increased relative abundances of *R. gnavus*, *C. bacterium*, *C. bolteae*, and *C. asparagiforme* were associated with better vitality, health change, and motivation scores. Decreased relative abundances of *F. prausnitzii* and *C. catus* were associated with worse emotional wellbeing scores, while decreased abundances of *R. inulinivorans* and *D. formicigenerans* were associated with improved motivation scores.

In the ME/CFS + IBS cases, decreased relative abundance of unclassified *Alistipes*, *D. longicatena*, and *R. inulinivorans* were associated with improved vitality, health change, and fatigue scores*.* Decreased relative abundance of *C. comes* and *Faecalibacterium* species were associated with worse fatigue scores and worse pain scores, respectively.

In ME/CFS without IBS cases, the increased relative abundance of *P. capillosus* was associated with worse vitality, emotional wellbeing, health changes and motivation scores. The relative abundances of *D. formicigenerans* and *C. scindens were associated* with improved motivation scores, similar to patterns observed in total ME/CFS.

Metabolic pathways predicted from bacterial metagenomic gene content revealed correlations between activity in specific pathways and clinical features. Decreased polyamine biosynthesis in both ME/CFS and ME/CFS + IBS cases was associated with worse physical function scores and increased fatigue. In ME/CFS + IBS cases, increased fucose and rhamnose degradation and increased threonin biosynthesis were associated with worse general wellbeing and pain scores, decreased phenylalanine and tyrosine biosynthesis, and decreased pyrimidine deoxyribonucleoside degradation were associated with worse general wellbeing, mental fatigue and pain scores. Increased sulfolactate degradation in ME/CFS without IBS was associated with better pain scores.

## Discussion

ME/CFS is associated with systemic inflammation and both GI and neurological disturbances [1]. Accordingly, we investigated relationships between microbiota, metabolic pathways, and plasma cytokine profiles in subjects with ME/CFS and matched controls. Metagenomic analysis and predictive selection revealed bacterial species whose relative abundance was associated with ME/CFS. Based on findings from TDA, LEfSe, and prediction models, *Faecalibacterium*, *Roseburia*, *Dorea*, *Coprococcus*, *Clostridium*, *Ruminococcus*, and *Coprobacillus* were strongly associated with ME/CFS; their combined relative abundance appeared to be predictive of diagnosis.

We cannot directly compare metagenomic results obtained here with others based on 16S rRNA analyses. Nonetheless, our findings replicate those of other groups in demonstrating intestinal dysbiosis in ME/CFS [12, 13, 22]. Fremont et al. found decreased abundance of several *Firmicutes* populations (such as *Roseburia*, *Synthrophococcus*, *Holdemania*, and *Dialister*) and an increased abundance of *Lactonifactor* and *Alistipes* [13]. Giloteaux et al. reported a reduction in the abundance of *Firmicutes* and differences from controls in representation of 40 bacterial species including *F. prausnitzii*, *Ruminococcus* spp, *Coprococcus* spp, *E. lenta*, and *C. aerofaciens* [12]. Our findings also demonstrate decreased *Faecalibacterium* species and increased *Alistipes* in ME/CFS as the strongest predictors for the disease.

The prevalence of IBS co-morbidity is high in individuals with ME/CFS (35–90%) [4–6]. The underlying link between these conditions and the directionality of the association remains to be addressed. ME/CFS and its underlying pathophysiology or the emotional responses to illness in these individuals may predispose ME/CFS sufferers to IBS. Anxiety (especially health anxiety) and depression are common in ME/CFS [23], and anxiety and depression increase the risk for IBS onset twofold [24]. Alternatively, the association between ME/CFS and IBS could arise as a result of overlapping pathophysiological mechanisms that contribute to the development of both syndromes. For example, infectious gastroenteritis caused by bacterial (*Campylobacter jejuni*, *Salmonella enterica*, *Shigella sonnei*, *Escherichia coli* 0157:H7), viral (norovirus), or protozoal (*Giardia lamblia*) pathogens increase the risk of post-infectious IBS [25]. ME/CFS is often reported to develop following an acute infectious illness, and post-infective fatigue states have been reported following bacterial, viral, and protozoal infections [26]. Acute giardiasis, for example, is associated with both increased risk of post-infective IBS (relative risk = 3.4 [95% CI 2.9–3.8]) and increased risk of post-infective chronic fatigue (relative risk = 4.0 [95% CI 3.5–4.5]) [27]. Finally, the association between these syndromes could derive from symptom overlap. Indeed, there is symptom overlap between IBS and other functional somatic syndromes, including ME/CFS and fibromyalgia syndrome [5]. IBS patients also have higher scores on the Fatigue Impact Scale than healthy individuals [28, 29].

TDA analysis revealed that IBS co-morbidity was a major driver of topological networks in our ME/CFS cohort. Decreased relative abundance of *Faecalibacterium* species, *R. obeum*, *E. hallii*, and *C. comes* was associated with IBS co-morbidity. The *Anaerostipes* genus was increased in ME/CFS + IBS, but not in ME/CFS without IBS. In contrast, the relative abundance of unclassified *Bacteroides*, *P. capillosus*, *E. lenta* (each increased) and *P. distasonis* (decreased) were identified as specific markers for ME/CFS without IBS. The relative abundance of *D. longicatena* was increased in ME/CFS without IBS but decreased in ME/CFS with IBS. Thus, whereas some differences in bacterial taxa in the overall ME/CFS cohort are driven by the high prevalence of IBS co-morbidity, others are specific to ME/CFS.

The decreased abundance of *Faecalibacterium* and *Coprococcus* species is associated with IBS-like symptoms, including colonic hypersensitivity, bloating, and GI discomfort in human and animal models [30, 31]. An altered microbiome is postulated to lead to increased gut permeability (“leaky gut”) and intestinal inflammation with gastrointestinal symptoms. Increased translocation of lipopolysaccharides (LPS) from gram-negative bacteria leads to autoantibody production, disruption of tight junctions, and both local gastrointestinal and systemic inflammation [12, 32, 33]. Prior findings demonstrating alterations in the microbiota of IBS patients were confirmed here by the strong association of these bacteria in ME/CFS individuals with IBS. Given the high rate of IBS co-morbidity in ME/CFS, such findings highlight the importance of considering IBS co-morbidity in studies evaluating the role of the microbiome in ME/CFS.

Metabolic pathways predicted from bacterial metagenomic gene content revealed additional alterations in ME/CFS and ME/CFS subgroups. Similar to our findings for differences in bacterial composition, differences in predicted bacterial metabolic pathways found in the total ME/CFS group were representative of aggregate findings associated with IBS subgroups. These results suggest that, as with bacterial taxa, some bacterial metabolic pathways may be uniquely altered in ME/CFS while others may be linked to IBS co-morbidity.

Enrichment in the pathway for vitamin B6 biosynthesis and salvage was the strongest predictor of ME/CFS as well as ME/CFS without IBS, suggesting that this association is independent of IBS. Reduced functional B-vitamin status has been reported in the ME/CFS patients; however, it is unclear whether such differences can be attributed to aberrant host or bacterial metabolic pathways [34]. Pyrimidine deoxyribonucleoside degradation and individual pathways linked to the TCA cycle are energy regulating pathways in host metabolism. Further highlighting the differences between ME/CFS with or without IBS, the predicted bacterial pathway of pyrimidine ribonucleoside degradation was enriched in ME/CFS without IBS (and in the total ME/CFS group) but was reduced in ME/CFS with IBS compared to controls. TCA and energy metabolism may influence the pathophysiology of ME/CFS through deficient adenosine triphosphate (ATP) production [35]. Intermediate metabolites linked to TCA cycles were identified as specific markers of ME/CFS in metabolomic analyses; however, it is unclear whether bacterial dysbiosis contributes to these host metabolic changes [36]. The metabolites and components of the urea cycle (such as AA and ammonia) are also reportedly altered in ME/CFS [36]. However, our results indicate that the majority of bacterial AA metabolic pathways that were associated with ME/CFS were only associated with the ME/CFS + IBS subgroup. Thus, if bacterial metabolic pathways contribute to these observed host metabolite changes, such changes could be restricted to the IBS subgroup.

Enriched pathways for the degradation of atrazine in ME/CFS were also found in our analyses and may be independent of IBS, as the predicted pathway of atrazine degradation was a biomarker of both ME/CFS without IBS and ME/CFS + IBS. Additional studies would be needed to determine whether atrazine, a chemical found in pesticides, is present in the GI tract of these individuals and is subject to degradation by these pathways.

The unsaturated FA biosynthesis pathway that was predicted to be reduced in all three ME/CFS groups is linked to energy homeostasis and basic components for several catabolic processes. The ratio of ω3/ω6 FAs and eicosapentaenoic acid/arachidonic acid are reduced in ME/CFS patients [37]. The lowered ω3 FA and altered ratio of mono- and polyunsaturated FAs are linked to pro-inflammatory responses and immune activation [38, 39]. The genes involved in mycolate biosynthesis, a bacterial pathway predicted to be reduced for ME/CFS without IBS in this study, are further linked to FA metabolic pathway initiation and metabolic processes [40]. The reduced representation of pathways for heme biosynthesis, as well as arginine and polyamine biosynthesis pathways are specific for ME/CFS cases with IBS co-morbidity. Arginine is a precursor of the production of nitric oxide (oxidative stress responses) and ammonia (urea cycle) metabolism [41]. Arginine is linked to increased energy and endurance, enhanced memory, and decreased intestinal inflammation via nervous system signaling [42]. While it has been demonstrated that bacteria in the gut microbiome play an important role in supplying vitamins to the host and that the gut microbiome has a profound influence on mammalian metabolites, additional studies will be needed to assess the relationship between the bacterial metabolic pathways identified here based on gene content, bacterial metatranscriptomics, and the metabolome in ME/CFS [43–46].

Specific bacterial species and linked metabolic pathways were correlated with ME/CFS disease score severity (vitality, mental fatigue and pain scores). Previous studies on changes in gut microbiome following exercise challenge in ME/CFS showed alteration within 72 h in ME/CFS subjects compared to baseline and to controls [22]. Our study did not find association between metagenomic data and reported post-exercise malaise/physical fatigue. However, our study was not designed to assess microbiome changes following exercise in ME/CFS patients.

Plasma cytokines did not define ME/CFS disease groups in our cohort. Previous studies demonstrated increased pro- and anti-inflammatory cytokine levels (such as TNFα) in plasma and cerebrospinal fluid of ME/CFS patients with short duration of the disease [47, 48]. The less robust findings of plasma immune molecule changes in the current cohort may be explained by the dearth of ME/CFS cases that had been ill just a short time.

## Conclusions

Our results confirm and extend previous work indicating intestinal dysbiosis in ME/CFS. We further demonstrate that patterns of dysbiosis vary with IBS co-morbidity. Future prospective studies should consider more detailed exploration of IBS subtypes, associated GI symptoms, and their relationship to ME/CFS dysbiosis. The identification of ME/CFS networks—characterized by specific profiles that integrate microbiota, metabolic pathways, and plasma immune molecules—may enable more accurate diagnosis and lead to insights that inform the development of specific therapeutic strategies.

## Methods

### Study population

Subjects included 50 cases and 50 controls from the Chronic Fatigue Initiative (CFI) Cohort [49] recruited at four sites across the USA who met the 1994 CDC Fukuda [17] and/or 2003 Canadian consensus criteria for ME/CFS [18]. Controls were frequency matched to cases on age, sex, race/ethnicity, geographic/clinical site, and season of sampling [50]. The Fukuda criteria require that subjects have persistent or relapsing fatigue for a minimum of 6 months and a substantial reduction in occupational, educational, social, or personal activities. In addition, subjects must have at least four of eight of the following symptoms, including sore throat, lymph node pain, muscle pain, joint pain, post-exertional malaise, headaches of a new or different type, memory and concentration difficulties, and unrefreshing sleep. The more restrictive Canadian criteria also require that the subject must have not only severe fatigue but also post-exertional malaise with loss of physical or mental stamina, nonrestorative sleep, disturbed sleep quantity or rhythm, arthralgia and/or myalgia, two or more neurocognitive manifestations, and autonomic, neuroendocrine or immune dysfunction.

All ME/CFS subjects (*n* = 50) completed standardized screening and assessment instruments including medical history and symptom rating scales, had a physical examination, and provided simultaneous fecal and blood samples. The fecal samples were collected by subjects 24 to 48 h prior to clinical visits, stored at −20 °C, and transferred to clinical site in styrofoam boxes with ice packs provided by study coordinators. Stool and plasma materials were shipped from clinical sites to the Columbia University laboratory site on dry ice and stored at −80 °C prior to processing. Controls (*n* = 50) of the earlier CFI cohort study [50] had been found to be free of self-reported ME/CFS or ME/CFS symptoms or other conditions deemed by the recruiting physician to be nonrepresentative of a healthy control population, including substance abuse in the prior year and any history of self-reported psychiatric illness; antibiotics in the prior 3 months; immunomodulatory medications in the prior year; and clinically significant findings on physical exam or screening laboratory tests. IBS co-morbidity was not part of the exclusion criteria of controls.

All participants provided informed written consent in accordance with protocols approved by the Institutional Review Board at Columbia University Medical Center.

### Clinical assessments

Clinical symptoms and baseline health status were assessed on the day of physical examination and biological sample collection from both cases and control subjects, using two surveys: the Short Form 36 Health Survey (SF-36) and the Multidimensional Fatigue Inventory (MFI). The SF-36 includes the following subject-reported evaluations about the current health status: physical and social functioning, physical and emotional limitations, vitality, pain, general health perceptions, and mental health change [51]. Each functional domain was transformed into a 0–100 scale, wherein a score of 100 is equivalent to maximum disability and a score of zero is equivalent to no disability. The MFI comprises a 20-item self-report questionnaire focused on general, physical, and mental fatigue, activity, and motivation [52]. The MFI score was converted to a 0–100-scale score to facilitate combination and comparison with data obtained using the SF-36 inventory.

IBS co-morbidity was based on subject-reported diagnoses of IBS on the medical history forms. The subjects were asked if they had been diagnosed with IBS by their physician. Additional questions on the severity of symptoms and bowel frequency were included. High BMI was defined as BMI >25 kg/m^2^ and normal BMI was defined as BMI <25 kg/m^2^. No subjects with a BMI below 18 kg/m^2^ (low BMI) were enrolled in our study.

### Shotgun metagenomic sequencing and bioinformatic analyses

SMS was carried out on DNA extracts obtained from the 100 fecal samples (50 cases and 50 controls). For Illumina library preparation, genomic DNA was sheared to a 200-bp average fragment length using a Covaris E210 focused ultrasonicator. Sheared DNA was purified and used for Illumina library construction using the KAPA Hyper Prep kit (KK8504, Kapa Biosystems). Sequencing libraries were quantified using an Agilent Bioanalyzer 2100. Sequencing was carried out on the Illumina HiSeq 4000 platform (Illumina, San Diego, CA, USA). SMS libraries from cases and controls were grouped into 10 different pools (10 individuals/pool). Each pool yielded an average of 350 million 100-bp, paired-end reads (mean = 7 Gb of sequence data per sample; median = 6 Gb). Raw SMS data were pre-processed using prinseq (v0.20.3) for end trimming and filtered to exclude low-quality and low-complexity reads. Adaptor sequences were removed using cutadapt (v 1.8.3). Human sequences were subtracted from the dataset using bowtie2 (v2.1.0) and using genomic, mitochondrial, and ribosomal sequences downloaded from NCBI. Bacterial composition (relative abundance) was obtained from raw sequencing data using Metaphlan (v1.7.8) software and processed by Qiime (v1.8). To evaluate overall microbiome differences, we used principal coordinate analysis based on the Bray-Curtis dissimilarity metric. Metabolic pathway analysis was carried out on host-subtracted sequences using Humann2 (v0.7.1) software.

### Immune profiling analyses

A magnetic bead-based 61-plex immunoassay (customized Procarta^TM^ immunoassay, Affymetrix) was used to measure plasma concentrations of immune molecules (Additional file 1: Table S3A). Case and control plasma samples were coded, randomized, and run in duplicate along with serial standards, buffer controls, and in-house human control plasma samples [53]. Mean fluorescence intensities of analyte-specific immunoassay bead sets were detected by flow-based Luminex™ 200 suspension array system (Luminex Corporation, Austin, TX) [54]. Cytokine concentrations were calculated by xPONENT (build 3.1.971.0) and Milliplex Analyst^TM^ software (v.5.1.0.0) using a standard curve derived from known reference concentrations supplied by the manufacturer. A five-parameter model was used to calculate final concentrations by interpolation. Values are expressed in pg/ml. Concentrations obtained below the sensitivity limit of detection (LOD) of the method were recoded to the mid-point between zero and the LOD for that analyte for statistical comparisons. Values obtained from reading of samples that exceeded the upper limit of the sensitivity method were further diluted and cytokine concentrations calculated accordingly. Feature scaling (data normalization) was used to standardize the range of cytokine values for the MeV heatmap [55], and log-transformation was used for network analysis.

### Topological data analyses

Metagenomic data including bacterial composition and inferred metabolic pathways, plasma immune profiles, and health symptom severity scores were integrated for topological data analysis (TDA) using the AYASDI platform (Ayasdi, Menlo Park, California). AYASDI represents high-dimensional, complex biological data sets as a structured 3-dimensional network [56]. Each node in the network comprises one or more subject(s) who share variables in multiple dimensions. Lines connect network nodes that contain shared data points. Unlike traditional network models where a single sample makes a single node, the size of a node in the topological network was proportional to the number of variables with a similar profile. We built a network comprised of 100 samples and 1358 variables (574 variables representing bacterial relative abundance at different taxonomic levels, 61 variables reflecting levels of each immune molecule in the assay, 586 variables representing metabolic pathways, 80 variables representing different ME/CFS fatigue, and other symptom score/health questionnaire items and information on co-morbidities; and demographic variables). All variables were weighted equally. Normalized correlation and variance-normalized Euclidean distance methods were used as the distance metric; a range of filter lenses (neighborhood lens 1 and 2, ME/CFS, and IBS diagnosis) was used to identify networks.

Standard statistical methods were applied to define the primary variables of these networks. Data were compared by nonparametric Kolmogorov-Smirnov (KS) tests to identify significant differences between networks.

### Statistical analyses

Between-group differences (ME/CFS, ME/CFS + IBS, ME/CFS without IBS, and controls) in microbial composition, fecal metabolic pathway expression, plasma immune molecules, and symptom severity were tested using the nonparametric Mann-Whitney *U* test. Benjamini-Hochberg FDR (false discovery rate) method was used to control the type I error rate at the 0.2 level [57]. Correlations between bacterial species and disease score were examined using nonparametric Spearman correlation.

Bacterial metagenomic and immune profiling assay data were used to develop a logistic regression model for prediction of the following binary response variables: the diagnostic groups ME/CFS, ME/CFS + IBS, ME/CFS without IBS, and controls. To eliminate potential multicollinearity, we used least absolute shrinkage and selection operation (LASSO) [58] and random forest (RF) [59] feature selection techniques to reduce high-dimensional data into a representative set of variables. Partial least squares (PLS) regression was used to determine the contributions of individual variables to the latent variable that explained the largest portion of the covariance. In-sample receiver operating characteristic (ROC) curves were plotted and area under the curve (AUC) was measured to compare models. To assess the predictive accuracy of the logistic regression models, random resampling cross-validation was performed with 1000 iterations. Data were randomly split into a training set (80%) and a test set (20%) within each iteration. AUC values, prediction error rates, false positive and negative rates were then averaged across iterations for all test sets. Sex, age, race, ethnicity, BMI, site, and season of sample collection were included in all statistical models as potential confounders.

Differences in the relative abundance of bacteria at all taxonomic levels were determined with linear discriminant analysis effect size [60], which couples tests of statistical significance with measures of effect size to rank the relevance of differentially abundant taxa [61]. Thus, the Kruskal-Wallis test identifies taxa that are significantly different in relative abundance among different classes, and the linear discriminant analysis (LDA) identifies the effect size with which these taxa differentiate the classes. For each LEfSe analysis, an alpha value of 0.05 for the Kruskal-Wallis test and a log-transformed LDA score of 2.0 were used as thresholds for significance. LEfSe analyses were used to evaluate differences among the fecal microbiome of the ME/CFS, ME/CFS + IBS, ME/CFS without IBS, and controls.

Data were analyzed and visualized with SPSS (IBM, NY), Matlab (R2013a, The Mathworks Inc., MA), Prism 7 (GraphPad Software, CA), BioVenn [62], and Circos [63] software. Genomic data analyzer (Multiple Experiment Viewer, MeV 4.8, MA) was used to define the clustering of metagenomic and immune profile data (with Spearman correlation and Euclidean distance metrics).

## Additional files

Additional file 1: **Table S1.** (A) TDA revealed significant bacterial and metabolic pathway profile differences in ME/CFS and ME/CFS + IBS but not in ME/CFS without IBS compared to control (the table shows the top most significant bacterial taxa, bacterial metabolic superpathways (SPWY) and individual bacterial metabolic pathways (IMPWY)). ME/CFS: myalgic encephalomyelitis/chronic fatigue syndrome, IBS: irritable bowel syndrome, KS score: Kolmogorov-Smirnov test, p: phylum, f: family, g:genus, s: species. (B) Fecal metagenomic and immune molecule profiles of ME/CFS networks based on topological data analysis. Bacterial composition, metabolic pathways, plasma immune molecules, and clinical data of ME/CFS patients separated into subgroups based on IBS co-morbidity and BMI. (ME/CFS: myalgic encephalomyelitis/chronic fatigue syndrome, IBS: irritable bowel syndrome, BMI: body mass index, CHO: carbohydrates, IL: interleukin). **Table S2.** Microbial composition differences between diagnostic groups (nonparametric Mann-Whitney *U* test, *p* < 0.05; false discovery rate is controlled at level 0.2 using Benjamini-Hochberg FDR method). (ME/CFS: myalgic encephalomyelitis/chronic fatigue syndrome, IBS: irritable bowel syndrome, k: kingdom, *p*: phylum, c: class, o: order, f: family, g: genus, s: species). **Table S3.** Predicted bacterial metabolic pathways analyzed by Humann2 (nonparametric Mann-Whitney *U* test, *p* < 0.01; false discovery rate is controlled at level 0.2 using Benjamini-Hochberg FDR method). (ME/CFS: myalgic encephalomyelitis/chronic fatigue syndrome, IBS: irritable bowel syndrome, TCA cycle: tricarboxylic acid cycle, FA: fatty acid, CHO: carbohydrates). **Table S4.** Immune molecules analyzed by 61-plex immunoassay. (ME/CFS: myalgic encephalomyelitis/chronic fatigue syndrome, IBS: irritable bowel syndrome). **Table S5.** Correlations of symptom severity scores with bacterial species abundance in ME/CFS and ME/CFS subgroups (ME/CFS + IBS and ME/CFS without IBS). Red indicates negative Spearman correlation; blue indicates positive Spearman correlation.

Additional file 2: **Figure S1.** Topological data analysis of ME/CFS group. (A) The ME/CFS cases clustered into four different groups based on IBS and BMI (normalized correlation metric and two lenses: IBS and BMI). (B) The mean relative abundance of individual bacterial species that discriminates between the ME/CFS clusters. The mean relative abundance is indicated by the surface area of the associated circle. The discriminative changes in bacterial composition are indicated by rectangles. (C–E) Association between measures of symptom severity based on SF-36 and MFI questionnaire items and ME/CFS subgroup-associated networks (shown in A) were evaluated with TDA. (C–D) Pain and physical disability were rated as more severe (color scale shown) in patients with ME/CFS + IBS who had a high BMI (indicated by ovals). (E) General fatigue rankings showed greater severity in patients with ME/CFS + IBS who had a high BMI and in ME/CFS without IBS patients with a high BMI (indicated by an oval) compared to other groups. Dots that are not connected in networks represent outliers. **Figure S2.** Plasma immune molecule profiles of ME/CFS and controls subjects. Heatmap showing results of unsupervised hierarchical clustering based on the Euclidean distance of plasma immune molecule concentrations (normalization with feature scaling). The normalized concentration of immune molecules is indicated by a color scale (below heatmap) that ranges from green (low value) through black to red (high value). The diagnostic group corresponding to each sample is shown in the bar below the heatmap where red = ME/CFS + IBS, blue = ME/CFS without IBS, and gray = controls. (Note that immune profiles show no clear relationship with diagnostic groups.)

## Abbreviations

BMI: Body mass index
FDR: False discovery rate
IBS: Irritable bowel syndrome
LASSO: Least absolute shrinkage and selection operation
LDA: Linear discriminant analysis
LEfSe: Linear discriminant analysis effect size
ME/CFS: Myalgic encephalomyelitis/chronic fatigue syndrome
MFI: Multidimensional fatigue inventory
PCoA: Principal coordinate analysis
PLS: Partial least squares
RF: Random forest
ROC AUC: Receiver operating characteristic and area under the curve
SF-36: Short Form 36 Health Survey
SMS: Shotgun metagenomic sequencing
TDA: Topological data analysis
