# Supplementary material for: Fecal metagenomic profiles in subgroups of patients with myalgic encephalomyelitis/chronic fatigue syndrome
Source: Microbiome. 2017 Apr 26;5:44. doi: 10.1186/s40168-017-0261-y (PMC5405467; doi:10.1186/s40168-017-0261-y)
Supplement: Supplementary file 2 — Figure S1. Topological data analysis of ME/CFS group. (A) The ME/CFS cases clustered into four different groups based on IBS and BMI (normalized correlation metric and two lenses: IBS and BMI). (B) The mean relative abundance of individual bacterial species that discriminates between the ME/CFS clusters. The mean relative abundance is indicated by the surface area of the associated circle. The discriminative changes in bacterial composition are indicated by rectangles. (C–E) Association between measures of symptom severity based on SF-36 and MFI questionnaire items and ME/CFS subgroup-associated networks (shown in A) were evaluated with TDA. (C–D) Pain and physical disability were rated as more severe (color scale shown) in patients with ME/CFS + IBS who had a high BMI (indicated by ovals). (E) General fatigue rankings showed greater severity in patients with ME/CFS + IBS who had a high BMI and in ME/CFS without IBS patients with a high BMI (indicated by an oval) compared to other groups. Dots that are not connected in networks represent outliers. Figure S2. Plasma immune molecule profiles of ME/CFS and controls subjects. Heatmap showing results of unsupervised hierarchical clustering based on the Euclidean distance of plasma immune molecule concentrations (normalization with feature scaling). The normalized concentration of immune molecules is indicated by a color scale (below heatmap) that ranges from green (low value) through black to red (high value). The diagnostic group corresponding to each sample is shown in the bar below the heatmap where red = ME/CFS + IBS, blue = ME/CFS without IBS, and gray = controls. (Note that immune profiles show no clear relationship with diagnostic groups.) [file 40168_2017_261_MOESM2_ESM.pptx]

## Slide 1
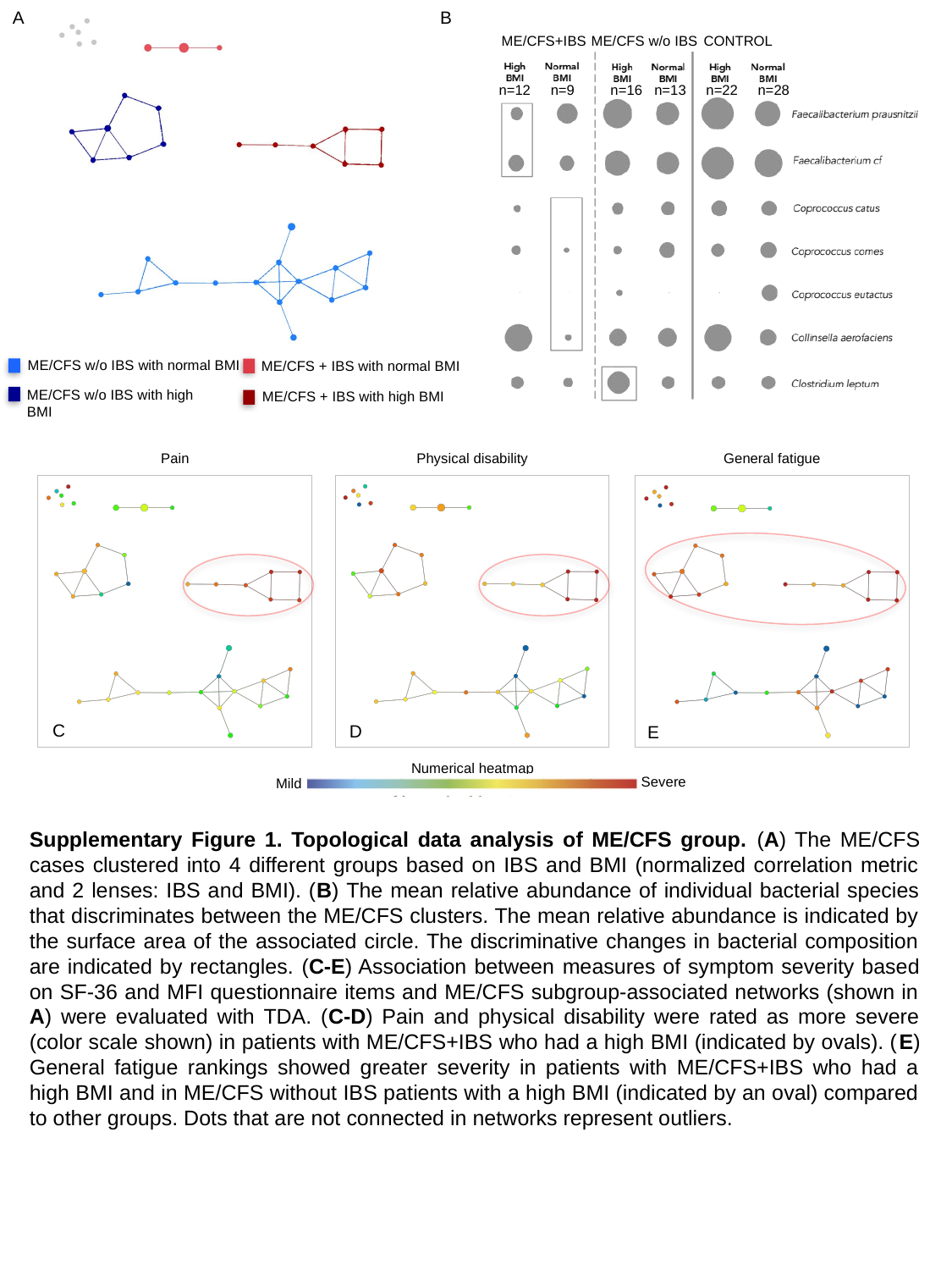

A
B
ME/CFS+IBS
ME/CFS w/o IBS
CONTROL
n=12 n=9 n=16 n=13 n=22 n=28
ME/CFS w/o IBS with normal BMI
ME/CFS + IBS with normal BMI
ME/CFS w/o IBS with high BMI
ME/CFS + IBS with high BMI
Pain
Physical disability
General fatigue
C
D
E
Numerical heatmap
Severe
Mild
Supplementary Figure 1. Topological data analysis of ME/CFS group. (A) The ME/CFS cases clustered into 4 different groups based on IBS and BMI (normalized correlation metric and 2 lenses: IBS and BMI). (B) The mean relative abundance of individual bacterial species that discriminates between the ME/CFS clusters. The mean relative abundance is indicated by the surface area of the associated circle. The discriminative changes in bacterial composition are indicated by rectangles. (C-E) Association between measures of symptom severity based on SF-36 and MFI questionnaire items and ME/CFS subgroup-associated networks (shown in A) were evaluated with TDA. (C-D) Pain and physical disability were rated as more severe (color scale shown) in patients with ME/CFS+IBS who had a high BMI (indicated by ovals). (E) General fatigue rankings showed greater severity in patients with ME/CFS+IBS who had a high BMI and in ME/CFS without IBS patients with a high BMI (indicated by an oval) compared to other groups. Dots that are not connected in networks represent outliers.

## Slide 2
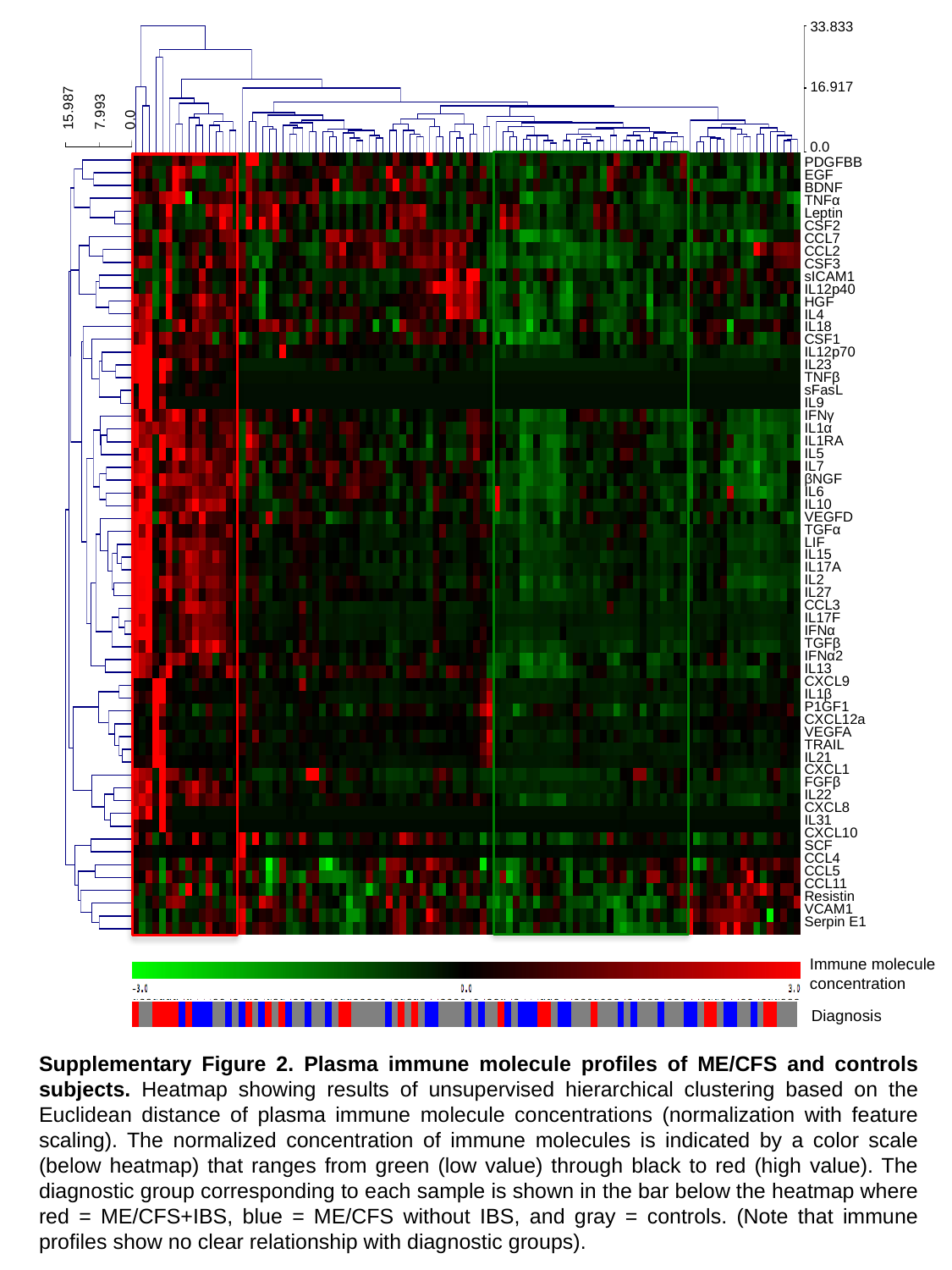

33.833
16.917
15.987
7.993
0.0
0.0
PDGFBB
EGF
BDNF
TNFα
Leptin
CSF2
CCL7
CCL2
CSF3
sICAM1
IL12p40
HGF
IL4
IL18
CSF1
IL12p70
IL23
TNFβ
sFasL
IL9
IFNγ
IL1α
IL1RA
IL5
IL7
βNGF
IL6
IL10
VEGFD
TGFα
LIF
IL15
IL17A
IL2
IL27
CCL3
IL17F
IFNα
TGFβ
IFNα2
IL13
CXCL9
IL1β
P1GF1
CXCL12a
VEGFA
TRAIL
IL21
CXCL1
FGFβ
IL22
CXCL8
IL31
CXCL10
SCF
CCL4
CCL5
CCL11
Resistin
VCAM1
Serpin E1
Immune molecule
concentration
Diagnosis
| 2 | 0 | 0 | 2 | 2 | 2 | 2 | 1 | 2 | 1 | 1 | 1 | 0 | 0 | 1 | 0 | 1 | 2 | 0 | 1 | 2 | 0 | 2 | 1 | 0 | 0 | 1 | 0 | 0 | 1 | 0 | 2 | 2 | 0 | 0 | 0 | 0 | 0 | 1 | 0 | 2 | 0 | 2 | 0 | 1 | 1 | 0 | 0 | 0 | 0 | 1 | 0 | 1 | 0 | 0 | 2 | 1 | 0 | 1 | 1 | 1 | 2 | 2 | 0 | 1 | 1 | 0 | 0 | 0 | 2 | 0 | 0 | 0 | 1 | 0 | 1 | 0 | 0 | 0 | 1 | 0 | 0 | 0 | 1 | 1 | 0 | 2 | 2 | 0 | 1 | 1 | 0 | 0 | 1 | 0 | 2 | 2 | 0 | 0 | 0 |
| --- | --- | --- | --- | --- | --- | --- | --- | --- | --- | --- | --- | --- | --- | --- | --- | --- | --- | --- | --- | --- | --- | --- | --- | --- | --- | --- | --- | --- | --- | --- | --- | --- | --- | --- | --- | --- | --- | --- | --- | --- | --- | --- | --- | --- | --- | --- | --- | --- | --- | --- | --- | --- | --- | --- | --- | --- | --- | --- | --- | --- | --- | --- | --- | --- | --- | --- | --- | --- | --- | --- | --- | --- | --- | --- | --- | --- | --- | --- | --- | --- | --- | --- | --- | --- | --- | --- | --- | --- | --- | --- | --- | --- | --- | --- | --- | --- | --- | --- | --- |
Supplementary Figure 2. Plasma immune molecule profiles of ME/CFS and controls subjects. Heatmap showing results of unsupervised hierarchical clustering based on the Euclidean distance of plasma immune molecule concentrations (normalization with feature scaling). The normalized concentration of immune molecules is indicated by a color scale (below heatmap) that ranges from green (low value) through black to red (high value). The diagnostic group corresponding to each sample is shown in the bar below the heatmap where red = ME/CFS+IBS, blue = ME/CFS without IBS, and gray = controls. (Note that immune profiles show no clear relationship with diagnostic groups).
